# Specific mutations in the genes of MC1R and TYR have an important influence on the determination of pheomelanin pigmentation in Korean native chickens

**DOI:** 10.5455/javar.2021.h511

**Published:** 2021-06-20

**Authors:** In Sik Nam, Min Gee Oh, Myoung Soo Nam, Woan Sub Kim

**Affiliations:** 1 Research Center for Environment Friendly and Quality Livestock Production Technology, Hankyong National University, 327, Jungang-ro, Ansung, Gyeonggi-do, 17579, Republic of Korea; 2 School of Animal Life Convergence Science, Hankyong National University, 327, Jungang-ro, Ansung, Gyeonggi-do, 17579, Republic of Korea; 3 Department of Animal Bio-system Science, College of Agriculture and Life Science, Chungnam National University, Daejeon 34134, Republic of Korea

**Keywords:** *TYR*, *MC1R*, melanin, chicken, plumage pigmentation

## Abstract

**Objective:**

The *TYR* (Tyrosinase) and *MC1R* (Melanocortin 1 receptor) genes are recognized as important genes involved in plumage pigmentation in Korean native chickens. Specifically, most color patterns in chicken result from differential expression of the *TYR* gene. In this study, the co-segregation of the pigmentation and sequence of the *TYR* and *MC1R* genes was investigated through intercrosses between red (R1q1), red with black and black plumage color types of native Korean chickens.

**Materials and Methods:**

Using DNA, RNA, and tissue by plumage color of each Korean native chickens, the role of major genes in pigmentation of pheomelanin was evaluated. Reverse transcription polymerase chain reaction, sequencing, western blot, and immunohistochemical were performed to determine the effect of *TYR* and *MC1R* genes on plumage pigmentation in Korean native chickens.

**Results:**

The KCO line (Korean chicken Ogol: Black-line) with an EEC _ genotype exhibited black feathers, whereas red and red mixed with black chicken with EeC genotype exhibited white feathers. There were notable differences between the base sequences of *MC1R* and *TYR* in three Korean chicken breeds, with the highest variation in *TYR*. Perhaps this is the key characteristics of Korean chicken. Further, we analyzed the expression patterns of MC1R and *TYR* genes in each type of Korea native chicken and observed that *TYR* expression was high in feather follicle (R1q2) of KCO tissue. However, native red (Korean chicken red) and native red with black (Korean chicken red dark) chickens have increased *TYR* expression in the tissue. However, the expression of *MC1R* was much different from that of *TYR*.

**Conclusion:**

In this study, our results suggest that the differences in position and *TYR* expression levels exert more influence on plumage pigmentation in native Korean chicken breeds than changes in *MC1R* expression levels.

## Introduction

The number of chicken varieties raised in Korea has rapidly decreased since 1952 with a number of improvements. The livestock chickens originated from Red Jungle Fowl (*Gallus gallus*) around 5400 BC [1,2]. There are various hypotheses about domestication of chickens. Melanin pigmentation determining the feather color of a chicken depends on sex differentiation and geographic location, and a complex association of melanin formation mechanisms and many genetic variations of *MC1R* (melanocortin 1 receptor) genes has been previously reported [3,4]. In particular, variation in the E region of chromosome 1 containing the locus of *MC1R* gene (R1q5) is an important factor in determining chicken feather color [3,5]. Extension genotype locus (the E locus in birds) encoding *MC1R* is also common in some mammalian species [6-8]. Korean native chickens have been endangered due to continuous improvement since 1952 [9]. Therefore, genetic mechanism analysis of Korean native chickens and the influence of major genes is very important. In the process of domestication of Red Jungle Fowl (*G. gallus*), the ancestor of chickens, various feather colors were produced due to local influences and environmental changes. Studies of genes related to plumage colors may provide major gene markers for breed-line identification; however, available genetic data on Korean native chickens is limited. The native chickens include all the indigenous species generated through interbreeding between Korean native chickens and foreign-introduced chicken varieties, which settled in the area for a long period of time, as well as native species with pure blood [10,11].

Recent studies have identified genotypes of Korean chickens using next generation sequencing technology, verifying phenotypes for H-types with brown color and L-types with black lines, but lack research on exact gene mechanisms [12]. Up until now, the most common approach to identify and group Korean chickens is via assessment of *MC1R* expression to determine the levels of pheomelanin or eumelanin in relation to chicken color [13,14]. However, an effective method to identify the color pattern of Korean native chickens is not yet available. Therefore, this study analyzes the nucleotide sequence variations in DNA based on the results of genotypic changes of *MC1R* [15] and *TYR* (tyrosinase) [16] in three domestic chickens from Gyeonggi-do, Korea. Moreover, we analyzed the differences of gene expression patterns in chicken feathers to establish basic data on the differences in feather colors of Korean chickens.

## Materials and Methods

### Collection of animal samples

**Figure 1.**
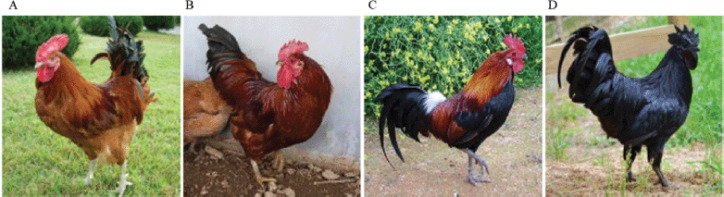
Classification of plumage color of Korean native chickens. A: KWC (Korean wild chicken), B: KCR (Korean native chicken Red), C: KCRD (Korean native chicken red with black), D: KCO (Ogol ; Korean native chicken black) [28].

For this study, black (KCO: Korean chicken Ogol), native red with black (KCRD: Korean chicken red dark) and native red (KCR: Korean chicken red) chickens, which are pure-blood lines raised at the National Institute of Animal Science (KOR), were selected and used in the experiment (Animal Experimentation Permission Number: 2020-3) (Fig. 1). The phenotypic profiles of developmental changes for three different patterns were observed at hatch and at 16 weeks of age. 10 chickens were randomly selected from each blood-line groups according to Kang’s research method, and samples were collected by slaughter [10]. After plumages were removed from each sample to extract deoxyribonucleic acid (DNA), ribonucleic acid (RNA), proteins and tissues, tissues including the dermis and epidermis of the back were collected.

### Phenotype and genotype of plumage colors

Genetic lucus type were analyzed by the polymerase chain reaction (PCR) amplification and single nucleotide polymorphism of both *TYR* (GenBank: DQ118701/DQ118702) and *MC1R* DNA (GenBank: AY220303, AY220304, and AY220305). The segregation of plumage pigmentation and genetic polymorphisms in *TYR* and *MC1R* genes was randomly analyzed across three phenotypes. We designed a pair of primers for *MC1R* genotyping analysis by PCR-RFLP using BalI restriction sites; different pairs of primers are designed for *TYR* genotype analysis [16] (Table 1).

### MC1R and TYR gene sequencing

The *MC1R* and *TYR* genes were amplified by PCR from the DNA of each sample groups and then separated by electrophoresis on a 2% agarose gel, and the amplicons were purified using the QIAamp DNA Kit (QIAGEN, Valencia, CA). The purified target DNA samples was sequenced using ABI 3100 Sequencer (Applied Biosystems, Foster City, CA), and the nucleotide sequence was analyzed by the Sequencing Analysis Version 3.3 (Applied Biosystems, USA).

**Table 1.** Primer for sequencing and real-time RT-PCR analysis.

No.	Primer name	Sequences (5' to 3')
1	MC1R-gF_1_	GCCATCCTCAAGAACAGGAA
2	MC1R-gR_1_	GCAGATGAGCATGTCGATGA
3	TYR-CC-F_1_	CAAAACCATAAATAGCACTGGAAATAG
4	TYR-mL-F_1_	CCTCTGGCTCTATTTGACTACACAGT
5	TYR-R_1_	TTGAGATACTGGAGGTCTTTAGAAATG
6	MC1R-qF_2_	GCCCTTCTTCTTCCACCTCAT
7	MC1R-qR_2_	GCTCCGGAAGGCATAGATCA
8	TYR-qF_2_	TGGTTTGCATAATGCCCTTCA
9	TYR-qR_2_	AACCACCGCTCAAAAATGCT
11	β-actin F	GAGAAATTGTGCGTGACATCA
12	β-actin R	CCTGAACCTCTCATTGCCA

F = forward; R = reverse, 1Primers for genotyping, 2Primers for real time RT-PCR.

### Complementary DNA (cDNA) synthesis and relative quantitative reverse transcription polymerase chain reaction (RT-PCR)

The total messenger RNA (mRNA) was extracted from the chicken tissues according to the TRIzol reagent (Invitrogen, Carlsbad, CA) method, and after purification using DNAase (Ambion, Austin, TX), cDNA was synthesized using Super-Script II (Invitrogen, Grand Island, NY). The RNA primers used for real time RT-PCR are shown in Table 1. RT-PCR amplification of mRNA genes were analyzed using the SYBR RT-PCR kit (TaKaRa, Shiga, Japan). Results were analyzed using cycle thresholds (Ct) using Rotor-Gene Real-Time Software 6.0 (BIOER, Tokyo Japan) to evaluate semi-log amplification plots. Finally, relative gene expression patterns were analyzed with β-actin mRNA expression levels as control groups (2-ΔΔCt method).

### Western blot analysis

To extract the total protein from each sample, the PRO-PREPTM kit (American Intron Biotechnology) was used. After, the total amount of each protein was quantified under the guidelines of Bradford Protein Analysis (Bio-Rad, CA, USA) and protein samples were kept at −80°C until they were used in the analysis. 30 μg of total protein extracted from each sample was separated on a 13% sodium dodecyl sulphate-polyacrylamide gel and transferred to a Immunoblot polyvinylidene fluoride membranes (Bio-Rad, USA). It was then detected using secondary antibody (anti-rabbit and/or anti-mouse secondary antibody; diluted 1:5,000; Abcam, MA) after inducing antigen antibody reactions using *MC1R* (diluted 1:1,000; TA308794, OriGene, USA, MD), *TYR* (diluted 1:1,000; AB6211, Abcam, UK, Cambridge) and β-actin (diluted 1:5,000; AB49900, Abcam) in the membrane where the protein was transferred. The membrane was then fluorescently reacted with ECL and analyzed after 1–5 min of exposure in diagnostic films.

### Immunohistochemistry

The paraffin sections were de-paraffinized in a xylene (Polyclear solvent; Polysciences, Warrington, PA), and antigen unmasking step were performed with 10 mm sodium citrate (pH 6.0). It was then detected using secondary antibody after inducing antigen antibody reactions using *MC1R* and *TYR* in each tissue sections for 1 h at room temperature. After antigen-antibody reaction, protein expression was detected with ABC reagent (Vactor, USA) and diaminobenzidene according to the manufacturer’s instructions. Counter staining to clearly confirm protein expression was performed using Harris hematoxylin (Fisher, Pittsburgh, PA, USA). Afterwards, the section slides were sealed with Permount (Fisher) and analyzed under an optical microscope.

### Statistical analysis

All the analysis results were repeated more than three times, and statistical significance was analyzed using SAS (Statistical Analysis System Institute, Version 9.4, Cary, NC). The data are represented by an average ± SD, and the significant difference between sample groups was determined at *p* < 0.05.

## Results

### Phenotypic and genotypic evaluation

The comparison results of Korea’s KCO, KCR, and KCRD breed-lines are shown in Table 2. The plumage color of the Korean native chickens was classified by phenotypes. Expectably, there was no apparent sexual dimorphism for the color of the Korean native chicken’s plumage. As a result of *MC1R* / *TYR* gene analysis, the pigmentation phenotype of the feathers of the group with the “EEC_” genotype in Korean chickens showed a black pattern, and the pigmentation phenotype of the feathers of the group with the “E_Cc” genotype was belonged to red and red with black.

### MC1R genetic distance of each group

**Figure 2.**
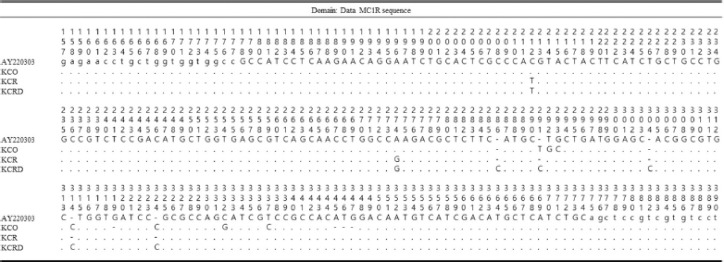
The identified SNP positions and haplotypes using *MC1R* gene control region in Korean native chicken.

The results of analyzing the base sequence of *MC1R* through DNA sequence analysis are shown in Figure 2. *MC1R* in AY220303 has 945 bp, KCO has 953 bp, KCR has 945 bp, and KCRD has 953 bp nucleotide sequences. A total of 11 nucleotide sequence variants exist between each group, and KCR and KCRD have 212 (C/T) and 274 (A/G) regions in which there is an existence of nucleotide variations at the same time. In addition, nucleotide added with KCO and KCRD are 314 (-/C) and 324 (-/C). However, unlike other groups, KCO has nucleotide variation in the intervals of 291 (-/T), 292 (T/G), 293 (G/C), 332 (C/G), and 337 (T/C), also nucleotide from 245 to 247 were removed. In addition, there was no significant difference between the base sequences, but KCO and KCRD had the most C-based sequence (36%) compared to other groups.

**Table 2.** Plumage color and *MC1R*­*TYR* genotype distributions in Korean native chickens.

Penotypin	*MC1R*–*TYR* genotype				
	EECC	EeCC	EECc	EeCc	Total
KCO: Black	7		3		10
KCR: Red with brown		7		3	10
KCRD: Red with yellow and black		6	1	3	10

### Differences in TYR nucleotide sequences among each groups

**Figure 3.**
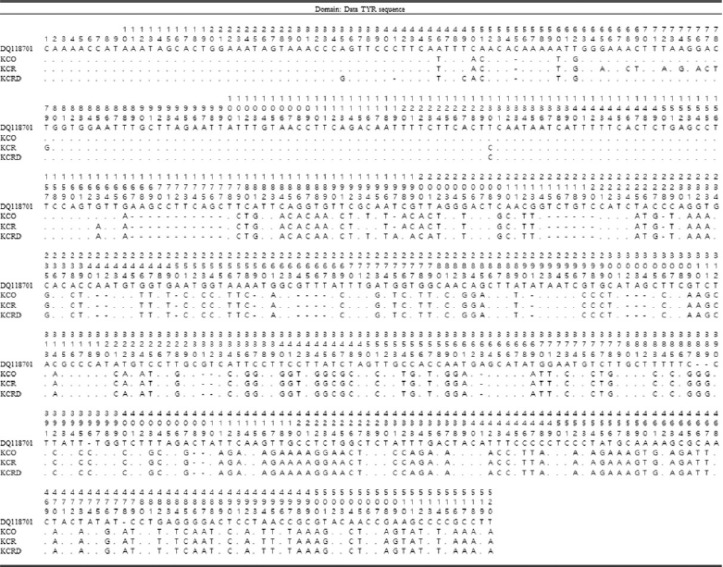
The identified SNP positions and haplotypes using *TYR* gene control region in Korean native chicken.

Figure 3 shows the results of analysis of *TYR* CC sequences in Korean native chickens. There were many differences the *TYR* gene when Korean species sequence was compared to the DQ11870 sequence, but all three Korean species had similar sequences among themselves. KCO showed a lot of difference from the nucleotide sequences of the other two species, KCR and KCRD, and there were differences in 200 nucleotide sequences. DQ118701 and KCO had the highest proportion of T (U) (31%) in the ratio of each base, and this result was different from base sequences of other species. In other words, Korean native species showed a lot of changes in the base of *TYR* CC relative to foreign species.

### Expression analysis of MC1R and TYR gene in each group

**Figure 4.**
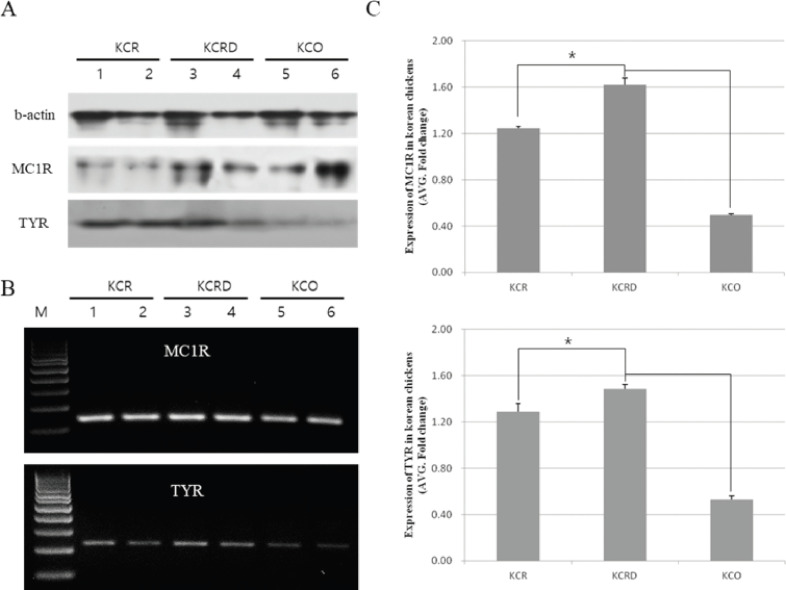
Gene expression analysis of *MC1R* and *TYR* in the skin samples of Korean native chickens. A: Western blot, B: QPCR analysis, C: Real-time PCR analysis. *Significant difference (*p *< 0.05).

**Figure 5.**
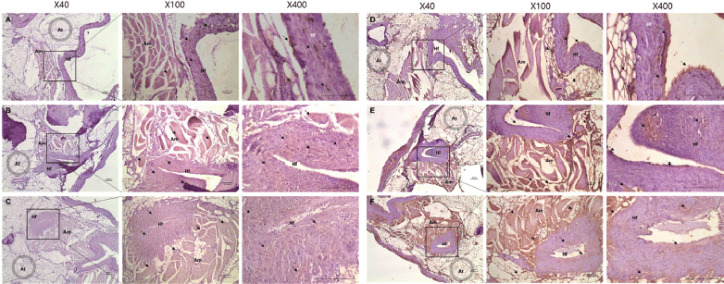
Localization of *MC1R*, *TYR* protein by skin samples in Korean native chickens. A figure magnification 40×, 100×, and 400×. A–C: MC1R expression, D–F: TYR expression, A, D: KCO, B, E: KCR, C,F: KCRD. The black arrow indicates protein expression position. Am: Arrector pili muscle, Hf: Hair follicles, At: Adipose tissue.

Gene expression analysis of *MC1R* and *TYR* in three Korean native chicken species is shown in Figures 4 and 5. Furthermore, we check the expression levels of *MC1R* and *TYR* at the mRNA level and observed that *MC1R* and *TYR* was highly expressed in KCRD, but was significantly lower in KCO. However, the expression patterns of proteins were different. *MC1R* expression was relatively high in KCO and relatively low in KCR. The expression patterns of *TYR* were relatively high in KCR and KCRD and relatively low in KCO (Fig. 4). Figure 3 shows the results of *MC1R* and *TYR* expression in the tissues of the outer feathers of each species. Expression of both genes was confirmed to be high in the hair follicles (Hf) and arrector pili muscle (Am) sections. In particular, the expression of *MC1R* was high in the melanocyte section present in the Hf of KCO. In the case of KCR and KCRD, the expression pattern was confirmed at the same site, but expression of KCRD was much higher than that of other groups. The expression of *TYR* was higher than that of *MC1R*. Unlike the expression of *MC1R*, the expression of TYR in Hf region was relatively high and the expression in KCRD was relatively lowly (Fig. 5).

## Discussion

Several studies showed that even though the genotypes of *MC1R* have the same phenotype, there are differences in protein combinations [16-18]. These results suggest that mutations or base substitutions of SNPs can affect various phenotypes of chicken feather color. In other words, all three Korean native chicken breeds have mutations in the *MC1R* gene, which may play an important role in changing chicken feather color [19-21]. In addition, similar variations were observed in species with red color, as Ellett et al. [19] findings indicate. Also, that the *TYR* was confirmed that the three species have a lot of differences compared to the existing nucleotide sequence, which can be said that the formation of *TYR* in the melanin metabolism process to control the increase or decrease of metabolism compared to *MC1R* [22]. That is, the expression of *TYR* seems to have a phenotypic effect on alterations of feather characteristics and some genes [22-24], suggesting that the expression of *TYR* may change depending on the mode of action of *TYR*, even *MC1R* expression is low. In particular, the mutation of the C locus located in intron 4 of TYR has several implications [25]. Moreover, it is possible to control the expression and variation of *TYR*, which seems to play a very important role in the determination of chicken feather color [16].

In our study, mutations in C locus were also identified, but other gene mutations were very high. Mutations in these genes were similar to the observation by Choi et al. [13], and the genetic variation of *TYR* and *MC1R* appears to be due to the environment and long-term settlement of Korean native chickens [25]. Based on these results, we found a very important difference in the analysis of the expression patterns of *TYR* and *MC1R* in this study, it can be confirmed that the TRY expression can affect the feather color even if the overall *MC1R* expression is low [22,26]. In our study, *MC1R* expression was high in the black system but low in the red line. The expression of *TYR* in the histological analysis was in contrast to the protein expression of *MC1R* and was high in the Hf of species with red color feathers. This suggests that feather color can be determined by the high expression of *TYR* in low stimulation of *MC1R* in Korean species in relation to *MC1R* and *TYR* [16,18]. However, the expression of *MC1R* and *TYR* genes in the black-line showed a lot of difference from previous studies. These results showed that *MC1R* gene was highly expressed, but *TYR* gene expression was relatively low, unlike previous studies on gene expression in black-line. This result is different from the fact that the high secretion of *TYR* increases the color of the dark hair, but unlike the red-colored species, the black-colored species has very high expression in Hf, Saha et al. [24] shows a similar pattern. In other words, our findings revealed genetic variation of *MC1R* and *TYR* and confirmed that there could be a very different pattern of protein expression in Korean chickens contrary to previous studies.

This study focused on the mutation of the existing *MC1R* and the deposition of feather color according to the region, but the results of other studies tended to focus on the large difference in mutation according to the environmental effect, which is a breeding program [24,27]. In addition, in terms of geographic ontogeny, the results of the association between *MC1R* and *TYR* as shown by Yang et al. [28] research suggest that even chickens of the same lineage may have different phenotypes. However, the results of this study suggest that *TYR* genetic variation may be formed according to *MC1R* mutations, and dynamic variation of *TYR* genetic sequence seem to play an important role in the regulation of plumage color in Korean native chicken. These results shows that variation in *MC1R* and *TYR* genes expressed within target tissues where feathers are formed can control the color determination of plumage color and the very complex properties of melanogenesis in Korean native chicken.

## Conclusion

In this study, we found that due to major gene mutations and melanogenesis mechanisms, differences in feather color in native chickens bred in each region may differ according to metabolic processes caused by unique mutations in the *MC1R* and *TYR* genes. In addition, this result is considered to be very important as a basic study to confirm that there are many differences in the color variation of native chickens in different countries and regions.

## List of Abbreviations

TYR: Tyrosinase; *MC1R*: Melanocortin 1 receptor; KCO: Korean chicken Ogol; KCRD: Korean chicken Red dark; KCR: Korean chicken Red; Am: Arrector pili muscle; At: Adipose tissue; Hf: Hair follicles; EEC, ECc, CC : E and C locus Genotype of TYR gene in chickens plumage color; KOR: Korea; RFLP: Restriction fragment length polymorphism; ECL: Enhanced chemiluminescence; SNPs: Single nucleotide polymorphism.

## References

[1] West B, Zhou BX (1989). Did chickens go north? New evidence for domestication. J Archaeol Sci.

[2] Crawford RD, Hunton P (1999). Origin, history, and distribution of commercial poultry. Poultry Production.

[3] Dunn PO, Whittingham LA, Pitcher TE (2001). Mating systems, sperm competition and the evolution of sexual dimorphism in birds. Evolution.

[4] Zhang GW, Liao Y, Zhang WX, Wu Y, Liu A (2017). A new dominant haplotype of MC1R gene in Chinese black plumage chicken. Anim Genet.

[5] Yang CW, Ran JS, Yu CL, Qiu MH, Zhang ZR, Du HR (2019). Polymorphism in MC1R, TYR and ASIP genes in different colored feather chickens. 3 Biotech.

[6] Klungland H, Vage DI, Gomez-Raya L, Adalsteinsson S, Lien S (1995). The role of melanocyte-stimulating hormone (MSH) receptor in bovine coat color determination. Mamm Genome.

[7] Kijas JMH, Wales R, Törnsten A, Chardon P, Moller M, Andersson L (1998). Melanocortin receptor 1 (MC1R) mutations and coat color in pigs. Genetics.

[8] Väge DI, Klungland H, Lu D, Cone RD (1999). Molecular and pharmacological characterization of dominant black coat colour in sheep. Mamm Genome.

[9] Sang BD, Kong HS, Kim HK, Choi CH, Kim SD, Cho YM (2006). Estimation of genetic parameters for economic traits in Korean native chickens. Anim Biosci.

[10] Kang BS (2010). Restoration of native chicken and industrialization of Korean chicken. Korea Poult Assoc.

[11] (2008). Breeding of domestic chickens and establishing certification standards.

[12] Eck SH, Benet-Pages A, Flisikowski K, Meitinger T, Fries R, Strom TM (2009). Whole genome sequencing of a single bos taurus animal for single nucleotide polymorphism discovery. Genome Biol.

[13] Choi JA, Lee JH, Jang HJ, Lee KT, Kim TH, Lee HJ (2014). Genetic variations of chicken TYR gene and associations with feather color of Korean native chicken (KNC). Korean J Poult Sci.

[14] Dávila SG, Gil MG, Resino-Talaván P, Campo JL (2014). Association between polymorphism in the melanocortin 1 receptor gene and E locus plumage color phenotype. Poult Sci.

[15] Xu JG, Xie MG, Zou SY, Liu XF, Li XH, Xie JF (2016). Interactions of allele E of the MC1R gene with FM and mutations in the MLPH gene cause the five-gray phenotype in the Anyi tile-like gray chicken. Genet Mol Res.

[16] Liu WB, Chen SR, Zheng JX, Qu LJ, Xu GY, Yang N (2010). Developmental phenotypic-genotypic associations of tyrosinase and melanocortin 1 receptor genes with changing profiles in chickens plumage pigmentation. Poult Sci.

[17] Oh JD, Lee KW, Seo OS, Cho BW, Jeon GJ, Lee HG (2010). Estimation of genetic characteristics and cumulative power of discrimination in Korean native chicken and Korean native commercial chicken. J Life Sci.

[18] Kim SH (2021). Identification of genetic association among different colors of Korean native chicken breeds through the RAPD-PCR method. J Anim Health Prod.

[19] Ellett AE, Okimoto R (2000). Melanocortin 1-receptor (MC1R-R) gene polymorphisms associated with chickens E locus alleles. Univers Student J Inquiry.

[20] Boichard M, Coville JL, Coquerelle G (2000). Polymorphism of the MC1R gene and feather color in the chicken: between breeds diversity and within family analysis.

[21] Ling MK, Lagerström MC, Fredriksson R, Okimoto R, Mundy NI, Takeuchi S (2003). Association of feather colour with constitutively active melanocortin 1 receptors in chicken. Eur J Biochem.

[22] Saha B, Singh SK, Sarkar C, Mallick S, Bera R, Bhadra R (2006). Transcriptional activation of tyrosinase gene by human placental sphingolipid. Glycoconj J.

[23] Murisier F, Guichard S, Beermann F (2007). The tyrosinase enhancer is activated by Sox10 and Mitf in mouse melanocytes. Pigment Cell Res.

[24] Saha B, Singh SK, Mallick S, Bera R, Datta PK, Mandal M (2009). Sphingolipid-mediated restoration of Mitf expression and repigmentation in vivo in a mouse model of hair graying. Pigment Cell Melanoma Res.

[25] Chang CM, Coville JL, Coquerelle G, Gourichon D, Oulmouden A, Tixier-Boichard M (2006). Complete association between a retroviral insertion in the tyrosinase gene and the recessive white mutation in chickens. BMC Genomics.

[26] Le Pape EL, Wakamatsu K, Ito S, Wolber R, Heraing VJ (2008). Regulation of eumelanin/pheomelanin synthesis and visible pigmentation in melanocytes by ligands of the melanocortin 1 receptor. Pigment Cell Melanoma Res.

[27] Hoque MR, Jin S, Heo KN, Kang BS, Jo C, Lee JH (2013). Investigation of MC1R SNPs and their relationships with plumage colors in Korean native chicken. Asian-Australas J Anim Sci.

[28] Yang CW, Ran JS, Yu CL, Qiu MH, Zhang ZR, Du HR (2019). Polymorphism in MC1R, TYR and ASIP genes in different colored feather chickens. 3 Biotech.

